# Collectively achieving primary health care and educational goals through school-based platforms: financing solutions for intersectoral collaboration

**DOI:** 10.3389/fpubh.2023.1241594

**Published:** 2023-11-28

**Authors:** Mackenzie Schiff, Ayan Jha, Dawne Walker, Eduardo Gonzalez-Pier

**Affiliations:** The Palladium Group, Washington, DC, United States

**Keywords:** primary health care (PHC), education, health-promoting schools, joint financing, co-financing, intersectoral

## Abstract

Despite abundant evidence demonstrating that improvements to health and education are positively correlated, and the importance of school-based platforms to achieve shared impacts, collaboration between ministries of health and education remains limited across low- and middle-income countries. Enhancing this collaboration is essential to realize mutually beneficial results, especially following the COVID-19 pandemic, which severely impacted health and education outcomes globally and highlighted the importance of resilient, domestically funded systems for delivering key social services including primary health care and education. We argue that the lack of an effective joint financing mechanism has hindered adoption of collaborative multisectoral approaches such as the WHO/UNESCO’s Health Promoting Schools (HPS) model. HPS is well-positioned to organize, finance, and deliver primary health care and education services through a school-based platform and strategy. Case studies from several low- and middle-income countries highlight the need to expand limited inter-ministerial collaborations to achieve cross-sectoral benefits and ensure sustainability of HPS beyond the lifecycle of external partners’ support. It is important to identify ways to widen the resource envelope for sector-specific activities and create efficiencies through mutually beneficial outcomes. This paper offers two pragmatic solutions: an inter-ministerial joint financing mechanism that starts with alignment of budgets but matures into a formal system for pooling funds, or a fixed-term co-financing mechanism that uses donor contributions to catalyze inter-ministerial collaborations. Achieving sustainability in these initiatives would require engaging the ministries of health, education, and finance; developing a common administrative, financial, and monitoring mechanism; and securing long-term commitment from all concerned stakeholders.

## Introduction

A sizeable and growing body of evidence from low-, middle-, and high-income countries shows that improvements to health and education are positively correlated (1–3). Education is one of the key social determinants of positive health behaviors and outcomes, and health has a marked impact on school attendance, educational attainment, and performance, especially at early ages (4, 5). Poor childhood health conditions have been associated with approximately 200–500 million lost school days per year, substandard learning outcomes, and subsequent dropouts (4). The past 3 years of the COVID-19 pandemic have severely impacted health and education outcomes in countries worldwide and have brought to focus the importance of resilient, domestically funded systems for the delivery of key social services including primary health care (PHC) and education. In the health sector, the pandemic not only amplified the clear need for universal PHC systems, but also – as a result of pandemic-induced economic recessions – intensified the resource constraints faced by most low- and middle-income countries (LMICs) to expand and strengthen PHC (6, 7). In the education sector, the abrupt closure of schools mandated under COVID-19 reinforced the importance of schools as platforms to influence children’s health and well-being positively and holistically. Evidence from countries around the world highlighted adverse effects such as large and persistent loss of learning, loss of access to school-based routine healthcare services and nutrition programs, and increased reports of children suffering from poor mental health. These impacts were disproportionately higher for children belonging to socio-economically disadvantaged families (8–10).

Countries across the world are expected to continue facing declining or slow growth and economic recovery through 2023 and strict domestic fiscal measures are likely to limit national spending in the social sector; the situation is especially worrying for LMICs (11, 12). Under such circumstances, innovative domestic financing approaches that look to pool resources from across allied sectors like health and education and focus on achieving efficiency may be particularly appealing to governments to continue delivering public goods including PHC and education. In this paper, we explore the feasibility of such financing mechanisms to support collaborative approaches between the health and education sectors, using the Health Promoting Schools (HPS) model advanced by the World Health Organization (WHO) and United Nations Educational, Scientific and Cultural Organization (UNESCO) as an illustrative model for intersectoral collaboration. First introduced over 25 years ago, HPS offers a school-based platform for joint health and education interventions, but the model has not been widely adopted by national governments or global development partners who continue to invest separately in PHC and educational programs in resource-poor settings (13). However, disruptions associated with COVID-19 have created an opportunity to revitalize focus on the HPS concept. WHO and UNESCO seized this critical juncture to relaunch HPS in June 2022, introducing a set of eight global standards which will be piloted in Botswana, Egypt, Ethiopia, Kenya, and Paraguay (13).

Despite the breadth of evidence demonstrating important links between the health and education sectors, we posit that the limiting factor preventing a widespread adoption of HPS has been the lack of a realistic, effective joint financing mechanism to fund the model. Indeed, most commonly, financing has been found to be the component which causes multisectoral mechanisms to fail (2, 15, 16). Our paper offers pragmatic solutions to that end.

## Health promoting schools: a mutual gains approach

HPS was introduced as a platform for the health and education departments to collaboratively develop a school-based program that targets concurrent improvements in health (both physical and mental), nutrition, and educational achievements of children (13). At the policymaking level, it envisions a “whole-of-government” approach as multiple ministries – such as the ministries of health; education; finance; social welfare; and women, children, and families – and levels of government (national and subnational) will need to become stakeholders in this process. At the operational level, HPS should ideally be a “whole-school approach,” which may require a reevaluation of school governance, teaching contents, and methods, and which should engage teachers, students, parents, health providers, and the community to create an environment that fosters good health and education. The benefits of this approach are summarized in Table 1.

**Table 1:** What can be achieved from investing in Health Promoting Schools?

Health and well-being	Education	Community	Government
Improved health-enabling environment in schools. Reduced risk factors for poor health outcomes within and outside school premises. Improved health and well-being of students, staff, and the wider community. Establishment of foundational knowledge, attitudes, and behavior to enhance health and well-being throughout the lifespan. Reduced inequities and inequalities in health outcomes.	Less inequality in educational outcomes. Less inequality in educational achievement. Improved school completion rates.	Sustained multisectoral collaboration that efficiently supports health, well-being, and education. Increased workforce capacity, social capital, and social cohesion.	Scaled-up health-promoting policies, plans, and activities. Decreased burden of disease in children and adolescents.

Recreated from (14).

Case studies from a purposive sample of eight LMICs^1^ across six WHO regions where the HPS model or some variation of health and education sector collaborations have been implemented highlight several key factors that enabled and hindered success (17). In Indonesia, for example, activities related to health in schools [known as *Usaha Kesehatan Sekolah* (UKS)] date back to 1980. While it is mandatory for all Indonesian schools to carry out some type of health promotion activities, implementation is inconsistent, which makes impact difficult to measure. However, education sector actors place very high value on winning Indonesia’s national competition for the best HPS, demonstrating the perceived importance of such initiatives. UKS is managed by the Joint Secretariat for Health in Schools, which is housed within the Ministry of Education and is responsible for joint planning and activities involving the ministries of health, education, religious affairs, and internal affairs. Development partners including WHO, United Nations Population Fund (UNFPA), and United Nations Children’s Fund (UNICEF) also support UKS activities. Notably, Indonesia funds UKS through national-level resource pooling, the funds from which are then distributed to and managed by districts, generally allocated by activity (for example, funding specifically for nutrition). Decentralized management can be a facilitating factor, as it allows for more context-specific interventions in each district and sharing of best practices across districts. However, the financial resources allocated remain insufficient, posing a significant obstacle to the successful scale-up and implementation of UKS. Other factors impeding success include inconsistent implementation support, lack of clear delineation of roles and responsibilities, frequent shifts in government, and lack of a national policy framework.

HPS-style initiatives have also been piloted in countries like Kenya and Zambia, demonstrating the potential impact of activities organized and financed jointly by the health and education sectors, with support from international development partners. In Kenya, the Ministry of Health and Ministry of Education, Science and Technology collaborated to implement a joint intervention with technical support from global implementing partners including Evidence Action, Innovations for Poverty Action, Children’s Investment Fund Foundation, and the END Fund. The two ministries and their partners collectively implemented a school-based deworming program serving children 2–14 years of age, regardless of their school enrollment status (18). In the first year alone, nearly six million children were dewormed in 112 participating districts. In each district, health and education department officials collaborated to organize training sessions for teachers, create community-level awareness, ensure smooth distribution of medicines to the schools, and monitor implementation. From the outset, the program’s alignment with existing government priorities, policies, and infrastructure was a key factor enabling the program’s success. The ministries also established a multi-level feedback loop through which personnel from both ministries were required to send monitoring forms to division leads, who sent these findings to district and county personnel, who in turn shared results with the national office. Thus, while this was essentially a targeted, vertical health intervention using public primary schools as treatment centers, it underlines the synergies that can be achieved between the health and education sectors through joint implementation. This example may also highlight a missed opportunity to formalize the collaboration through appropriate financing mechanisms and achieve sustainable, cross-sectoral benefits, which is the goal of the HPS strategy.

In Zambia, the Ministries of Education, Health, and Community Development and Social Services collaborated to implement a multisectoral school health and nutrition program with support from the USAID-funded CHANGES^2^ project (19). This was conceptualized as a precursor to larger integration of the health and education sectors in Zambian schools. Personnel from across the three ministries worked together to identify key health priorities, such as micronutrient deficiencies and HIV, and then designed the school health and nutrition intervention to address those issues. Stakeholders agreed on this cost-effective approach based on its projected positive impact on both health and education indicators. Robust, continuous monitoring throughout the pilot program was an important factor in its success. Even more significant was the Ministry of Education’s leadership and investment in developing the policies, systems, and tools necessary to implement the school-based health program alongside the Ministry of Health and implementing partners. The program contributed to significant improvement in child health outcomes, particularly the prevention of schistosomiasis (a common parasitic disease) and improved uptake of iron and vitamin A. Consequently, the program also led to increased enrollment, attendance, and academic performance in schools. In short, Zambia’s school-based health program depicts the cumulative cross-sectoral benefits of an HPS-type effort, but perhaps could have had broader impact and greater potential for sustainability if jointly financed by the involved ministries.

Based on these and similar country case studies, several key factors are seen to have enabled or hindered the success and long-term sustainability of HPS-style interventions. Insufficient financial resources are consistently one of the greatest barriers to implementation and sustainability of health and education programs (17–19). Even in Indonesia, where the government pools funding at the national level for HPS activities, the resources are insufficient to cover needs across all districts, and financing is generally allocated to certain activities rather than to the overall program. In Kenya and Zambia, ministries contributed in-kind resources (such as human resources or technology) which were valuable to implement the programs, but may not in themselves be sustainable without complementary financing. In these examples, the collaboration between the national ministries of health and education ensured allocation of resources to cover salaries and capacity building of school staff, operating expenses, and program monitoring and evaluation – a minimum necessity for the initiatives. Ideally, a national framework or strategy for HPS would be developed to create shared understanding. Clearly delineating the roles and responsibilities of all actors, as well as the benefits to all sectors or ministries involved, would also greatly improve collaboration. Additionally, it remains critical to consider the school and local community context while developing and incorporating health and educational activities in plans, policies, and teaching curriculum at the operational (school) level. In other words, the school and the community must become shared owners of the HPS program to drive sustainability.

## Financing the intersectoral HPS program

The above country experiences demonstrated strong administrative and technical collaboration between the ministries of health and education, yet lacked an effective or sufficient financing mechanism to ensure sustainability beyond the donor-funded and implementation partner-supported timeline. The short- and long-term success of collaborative efforts critically depend on identifying mechanisms that are acceptable to diverse stakeholders, including the ministries of education, health, and finance, as well as to donors and development partners (20). Though multisectoral pooled financing has not yet become ubiquitous in development programs, the literature identifies some mechanisms for jointly financing social sector collaborations, including those between education and health (16–18).

McGuire et al. conducted a systematic review of intersectoral joint financing mechanisms^3^ involving the health sector and identified two major approaches, which they refer to as “integrative” and “promotion” financing (16). Integrative approaches typically involve sectors that have some degree of overlap in their programming and targeted defined populations; however, it is notable that all case studies applying this approach were from high-income countries. In contrast, promotion financing approaches involved sectors with less overlap in programming and tended to be more intervention-centric; the Kenya and Zambia case studies above are examples. This macro-level distinction in financing approaches highlights the challenges of building long-term partnerships between social sector agencies (like health and education departments) in LMICs. Within these two broader categories, the authors identify a range of financing mechanisms. In high-income countries, intersectoral collaborations are often financed via pooling participating agencies’ budgets into one joint fund at the national or sub-national level; establishing joint working units with or without pooling of funds; or creating a grant-making system led by one stakeholder to fund intersectoral work. Less commonly, countries may form trusts or boards that completely integrate organizational and financial functions for the collaborative effort, or use penalties levied by one agency on another in case of delay in delivering their share of the collaborative services (without any actual organizational or financial integration). In sharp contrast, almost all case studies of intersectoral collaboration in LMICs use one of two financing mechanisms: either the participating ministries are individually responsible for financing and managing their share of activities (alignment of budgets) or each ministry offers in-kind contributions (such as human resources, infrastructure, or technology) to achieve the shared goals through the collaboration. In either case, the LMIC financing arrangements are reflective of the limited budgets and restricted financial autonomy of individual ministries. In some cases, these approaches also demonstrate limited confidence or trust between ministries and thus a weak level of partnership.

Separate from the above findings, co-financing offers another compelling option to support HPS implementation. Under a co-financing arrangement, donors and national government jointly fund a program (or specific parts of a program), with the government’s share gradually increasing over a defined period with the objective of reaching full domestic financing status. This mechanism has been widely used by Gavi, The Vaccine Alliance, to introduce new vaccines in LMICs, and is currently being considered to finance family planning commodities (21, 22). Given the likelihood that most intersectoral collaborations involving the ministries of health and education in LMICs would require varying degree of financial and technical support from donors and implementation partners, there is merit to fixed-term co-financing arrangements. This mechanism should work best when the Ministry of Finance is involved along with the ministries of health and education at the initial stages of agreement, so that the government’s financial commitment to sustain a collaborative HPS program is ensured.

In the context of global recovery from COVID-19-induced economic shocks and decreasing domestic fiscal space for development programs, especially in LMICs, we must use available evidence to improve and innovate in order to sustain the delivery of PHC and education. Box 1 presents our recommendations to finance initiatives like the HPS program – intersectoral collaborations through which the resource envelope for sector-specific activities is widened and efficiencies are created through mutually beneficial outcomes. We offer two pragmatic solutions: an inter-ministerial joint financing mechanism that starts with alignment of budgets but matures to a formal system for pooling funds, or a fixed-term co-financing mechanism that uses donor contributions to catalyze inter-ministerial collaborations. In proposing these solutions, we must also acknowledge the challenges that have hindered past efforts to promote and sustain intersectoral collaborations, as well as possible mitigative measures to address these obstacles.

Box 1 **Financing solutions for intersectoral collaboration for Health Promoting Schools program.**

1 **Intersectoral joint financing mechanism:**

Countries may follow a two-phase process to develop a sustainable joint financing mechanism between the ministries of health and education.

- The initial phase can involve an alignment of budget between the concerned ministries, especially if the collaboration focuses on limited, vertical interventions:
  ○ No pooling of financial resources, but participating ministries individually responsible for financing and managing their share of activities.
  ○ Critical to establish a common structure for management and accountability.
  ○ Helps develop working relationships and realize the value of achieving shared goals through maximizing efficiency.
- If successful, the collaborating ministries can move to a long-term plan of pooling budget at national or sub-national levels:
  ○ Creation of a common (joint) fund where financial contributions are pooled.
  ○ Essentially binds the ministries in a long-term (can be defined) commitment.
  ○ Requires involvement of the Ministry of Finance and (likely) legislative approval.

2 **Co-financing arrangement for a fixed term:**

A development partner (like USAID, World Bank, Global Partnership for Education, etc.) contributes funding for a set amount of time, with a clear roadmap to progressive country ownership as a precondition for funding.

- The donor and implementing partner must engage the Ministry of Finance along with the ministries of health and education to co-develop the agreement.
- Donor funding is intended to serve as a catalyst to move toward the country’s government taking financial and managerial responsibility for the Health Promoting Schools initiative.
- Critical to establish a common structure for management and accountability.
- While administratively complex, countries are incentivized to undertake intersectoral collaboration and a suitable period of time may be agreed upon for transitioning to full country ownership, thereby ensuring sustainability.

One reason ministries are often reluctant to formally collaborate is that they may perceive the risks of collaboration to outweigh potential benefits, or that decision-makers do not have a clear understanding of the anticipated benefits. Officials across ministries and levels may be especially hesitant to buy in to an idea that is perceived as primarily risky, for a variety of reasons including fears of job loss or multiplication of responsibilities without commensurate increase in team capacity (or pay), expectations that the program will fail, or uncertainty regarding their constituencies’ response to the initiative (16). Thus, it is important to demonstrate to stakeholders the anticipated value of their collaboration using compelling evidence that speaks to stakeholder priorities, such as the increase in enrolment in primary school that can be projected as a result of implementing a joint HPS mechanism. The two-phase joint financing approach we propose is designed to strengthen the enabling environment for inter-sectoral collaboration by gradually building support across all stakeholders.

Further, actors are often concerned that the inter-ministerial partnership will not be equitable in terms of financial investment, responsibilities, and alignment of sectoral priorities. A successful arrangement requires collaborative planning involving all relevant stakeholders during the design stages, setting clear terms of engagement, and developing a plan for sustainability (16). Equally important is the flexibility to adapt as needed throughout implementation as the operating context and the program itself evolves. Relatedly, inter-ministerial collaboration often fails due to a lack of trust or weak relationship between the sectors or ministries. It is critical that collaborating ministries engage regularly to build and maintain the relationship, and that all actors commit to continued communication and knowledge sharing. Ideally, ministries would create shared targets and indicators to track performance and progress to avoid falling prey to a lack of accountability and measurement.

Similarly, actors considering co-financing arrangements must be cognizant of certain well-documented pitfalls. Primarily, a complex financial arrangement like co-financing must be sensitive to a country’s evolving macroeconomic context over the period of the arrangement, so that the government can successfully comply with assuming increasing financial responsibility of the program and indeed achieve full ownership at the end (22). Analyses of data from countries which had co-financing agreements with Gavi (cost-sharing for new and under-used vaccines introduced with Gavi support) demonstrate that rising inflation and debt repayments have been major barriers to compliance. In many cases, countries have re-purposed monies from one health program or part of a program to meet their co-financing dues, thereby defeating the underlying goal of increased domestic resource mobilization. These experiences underscore the need to use sensitive metrics to determine a country’s economic status, which in turn guides the rate of increase in co-financing shares over the years – a principle adopted by the UNFPA Supplies Partnership in their co-financing of reproductive health commodities. Further, the need for the Ministry of Finance to be a co-signatory in co-financing agreements cannot be overstated, as this can help actors avoid defaulting on payments and ensure government’s commitment; however, finance ministries must be presented with compelling evidence of the efficiencies achieved through the proposed mechanism. The donor has a significant role to play in ensuring that the Ministry of Finance remains engaged and is incentivized to ensure new government health spending. Lastly, the success of this arrangement will depend on establishing a structured system to monitor progress, including forming joint coordination committees and establishing a dedicated co-financing account with the Ministry of Finance for budgetary transparency (23).

In making these recommendations, we consciously do not prioritize in-kind financing arrangements, which, though perhaps the most convenient option for ministries, are likely to be unsustainable as the stakeholders are not bound by any long-term commitments. However, we also do not undermine the utility of in-kind financing for certain vertical initiatives, or as a first step to develop a working relationship between ministries. Our recommended financing solutions look to create a pathway to sustaining collaboration beyond the limited period of external funding and technical support. Achieving this outcome requires engagement of the Ministry of Finance (in addition to health and education ministries), the development of a common administrative, financial, and monitoring mechanism, and codification of the long-term commitment from the concerned ministries. Once the intersectoral collaborations are established and operational, it is important for LMICs to continue exploring ways to secure and sustain additional domestic sources of funding – for example, through leveraging earmarked corporate social responsibility funds from large private sector organizations, especially those for whom children are an important target demographic.

## Conclusion

HPS offers an efficient, mutually beneficial model for the health and education ministries to collaborate at national and sub-national levels to better deliver essential PHC and education services through school-based platforms. The institutional nature of this program and its cumulatively derived cross-sectoral benefits should make HPS an attractive option to policymakers. Further, the post-COVID-19 world offers a unique opportunity to promote the revitalized HPS concept, given increased recognition during the pandemic period of the inter-connectedness of childhood health and education, and the importance of schools in achieving positive outcomes in these areas. In this context, we focus on the key question of financing such intersectoral collaborations – an issue that is often not directly addressed or defined, and which can be a major obstacle to long-term sustainability – and offer two potential mechanisms. However, for HPS to be successful in LMICs, there is a further need to leverage political will, strengthen domestic capacity for multisectoral accountability and monitoring, and persuade governments to collaborate internally by documenting success of pilot initiatives and conducting cost–benefit analyses to demonstrate the gains to all involved ministries.

## Data availability statement

The original contributions presented in the study are included in the article/supplementary material, further inquiries can be directed to the corresponding author.

## Author contributions

MS, AJ, DW, and EG-P: conceptualization, critical review, and finalization. MS and AJ: literature review, analysis, and drafting manuscript. All authors contributed to the article and approved the submitted version.

## Footnotes

^1^Bhutan and Indonesia (South-East Asia); Paraguay (Americas); Philippines (Western Pacific); South Africa and Senegal (Africa); Tunisia (Eastern Mediterranean); and Ukraine (Europe).

^2^Communities Supporting Health, HIV/AIDS, Nutrition, Gender, and Equity Education in Schools (CHANGES) program, funded by the United States Agency for International Development (USAID) with Creative Associates International as the primary contractor.

^3^McGuire et al. use the term “co-financing” instead. However, we consistently use the term “joint financing” to describe the system of pooling resources across national ministries, while we employ the term “co-financing” to refer to a financing mechanism where an external donor participates in collaboration with national government.
